# Transjugular Tricuspid Valve Implantation of Valve-in-Ring Bioprosthesis: Feasibility in a Preclinical, Pilot Trial

**DOI:** 10.5761/atcs.nm.24-00171

**Published:** 2025-04-04

**Authors:** Lishan Zhong, Qiuji Wang, Shuo Xiao, Junfei Zhao, Yingjie Ke, Zhaolong Zhang, Huanlei Huang

**Affiliations:** 1 Department of Pediatric Cardiology, Guangdong Provincial People’s Hospital, Guangdong Academy of Medical Sciences, Southern Medical University, Guangzhou, Guangdong, China; 2 Department of Cardiac Surgery, Guangdong Provincial People’s Hospital, Guangdong Academy of Medical Sciences, Southern Medical University, Guangzhou, Guangdong, China; 3 Department of Cardiovascular Surgery, The First People’s Hospital of Foshan, Foshan, Guangdong, China; 4 Department of Cardiovascular Surgery, Guangdong Provincial People’s Hospital’s Nanhai Hospital, The Second People’s Hospital of Nanhai District Foshan, Foshan, Guangdong, China; 5 Guangdong Cardiovascular Institute, Guangdong Provincial People’s Hospital, Guangdong Academy of Medical Sciences, Guangzhou, Guangdong, China

**Keywords:** tricuspid regurgitation, tricuspid annuloplasty, transcatheter tricuspid valve replacement, preclinical model

## Abstract

This preclinical study in a porcine model of recurrent regurgitation following tricuspid valvuloplasty aims to confirm the feasibility and safety of a novel transjugular tricuspid valve (TV) replacement device and to optimize the implantation procedure prior to first-in-human study. The novel device was implanted via a transjugular approach in a large white pig model (n = 2). No perivalvular leakage (PVL) or central tricuspid regurgitation (TR) was observed on post-operative echocardiography. The mean transvalvular gradient at 3 months follow-up was 1.69 ± 0.7 mmHg with mild central TR but no PVL. There was no right ventricular outflow tract obstruction, III atrioventricular block, device malposition, pericardial effusion, coronary artery compression, or myocardial infarction. This technique may be a promising option for patients after TV valvuloplasty and is ideal for high-risk patients undergoing open-heart surgery.

## Abbreviations

TR
: tricuspid regurgitation
TV
: tricuspid valve
TA
: tricuspid annuloplasty
PVL
: perivalvular leakage
RVOT
: right ventricular outflow tract
TTE
: transthoracic echocardiography
TTVD
: transcatheter tricuspid valve device

## Introduction

Tricuspid regurgitation (TR) affects more than 70 million people worldwide^1)^ and the risk of isolated TR surgery is high.^2)^ Therefore, in the 2020 American College of Cardiology/American Heart Association guideline, tricuspid valve (TV) surgery is considered a Class I indication for patients with severe TR at the time of left heart valve surgery, but a Class II indication for patients with moderate or less TR.^3)^ Various tricuspid annuloplasty (TA) techniques are used for the concomitant repair of TR. However, the cumulative incidence of TR recurrence is still relatively high, being 15.9%, 19.4% and 21.1% at 5 years for rigid ring, flexible band and suture annuloplasty, respectively.^4)^ In addition, in a substantial proportion of patients with previous left heart valve surgery, a redo thoracotomy is associated with higher mortality.^5)^ The minimally invasive surgical approach or transcatheter interventional approach is a better strategy for the treatment of TR in patients who have undergone TA; however, there is currently no transcatheter interventional device for use in patients with previous implantation of tricuspid rings.

The aim of this study is to develop a novel transcatheter tricuspid valve device (TTVD) (Shanghai Cingular Biotech Corporation, Shanghai, China) to adapt to the conditions of patients with recurrent severe TR who have undergone TA. In the present study, we describe the feasibility, safety, and preclinical porcine model outcomes of TV replacement via transjugular approaches on a beating heart.

## Materials and Methods

### Study design and oversight

The TTVD used in this study was developed jointly by Guangdong Provincial People’s Hospital and Shanghai Cingular Biotech Corp. This study was approved by the Ethics Review Committee of Guangdong Provincial People’s Hospital (approval number: 2019-720A-1), and all experiments were conducted in accordance with the National Laboratory Animal Care Regulations.

### Design of a novel interventional tricuspid valve

**Fig. 1:**
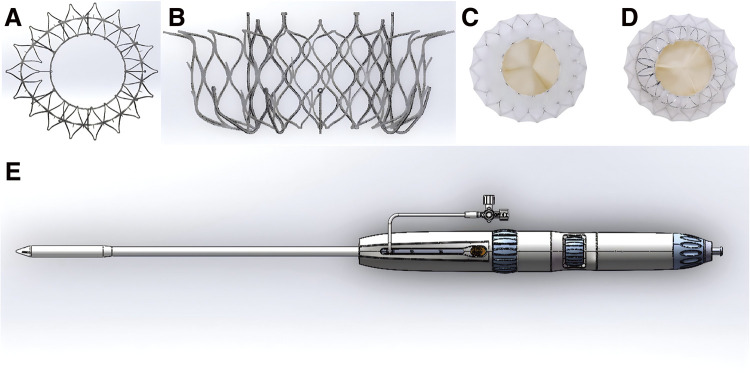
(**A**) Transjugular tricuspid valved stent and delivery system. Inflow (right atrial) view of the double-layered self-expanding nickel-titanium alloy stent. (**B**) Side view of the double-layered self-expanding nickel-titanium alloy stent. (**C**) Inflow (right atrial) view of valved stent. (**D**) Outflow (right ventricle) view of valved stent. (**E**) Lateral view of the transjugular delivery system.

The novel stent valve consists of a double-layered self-expanding nickel-titanium alloy stent, a bovine pericardial trileaflet prosthesis and a polytetrafluoroethylene braided skirt (**Fig. 1**). The inner stent has a unique connection structure that locks onto the transmitter and the outer layer of the stent has flared anchoring hooks, anchoring spikes and atrial end flanges. At the same time, we closed the groove between the inner and outer layers to prevent stagnation of blood flow in the groove and thus avoid thrombus formation. This also ensures that the structure of the inner layer of brackets is not affected by structural deformation of the outer layer. The stent has a soft overall shape that can be adapted to the lesion structure, allowing the stent to conform to the tricuspid annulus, effectively reducing perivalvular leakage (PVL). The diameter of the bovine pericardial valve is smaller than that of the tricuspid annulus, and the gap between the inner and outer stents does not interfere with annular retraction and reverse remodelling of the right ventricular structure. The adaptive polytetrafluoroethylene braided skirt does not compress the annulus in position, does not exert radial forces on the TA and further reduces PVL. The tricuspid device is currently available in four sizes: 36/27, 42/27, 48/29, and 54/29, and in the future we will develop the tricuspid device in sizes for extra-large or extra-small annular sizes.

### Delivery system

The transjugular delivery system consists of an outer and inner sheath with core (**Fig. 1D**). Delivery system diameter is 10 mm. The length of the working sheath is 30 cm, including the distal (6.5 cm) and proximal (23.5 cm) parts. The distal part of the outer sheath is flexible and can be bent 180° to adapt to different TA angles.

### Establishing TR model after tricuspid valvuloplasty

Large white pigs (n = 2) were used to create an animal model of TR with a tricuspid annulus prosthesis (Beijing Balance Medical Technology, Beijing, China). BalMedic Tricuspid Annuloplasty Ring is the first approved domestic annuloplasty ring in China. The ring is constructed of titanium core which having good formability. It can be shaped freely during surgery to better fit the anatomical structure of the tricuspid annulus. The BalMedic Tricuspid Annuloplasty Ring is not morphologically very different from other types of tricuspid annulus. The surgical procedure is briefly described here: Cardiopulmonary bypass was established after a median thoracotomy. The right atrium was incised, the tricuspid valve exposed and the anterior leaflet destroyed to create TR before a TA ring was placed according to the size of the tricuspid annulus, 25#Balance medical tricuspid ring was implanted in each pig. The right atrial incision was sutured, and the surgical incision was closed.

### Preoperative assessment for transjugular TTVR

Computed tomography (CT) and transthoracic echocardiography (TTE) were performed to measure the anatomical characterisation of the TA and to navigate the route through the jugular approach. CT and TTE were important for the animals to adjust the appropriate TV prosthesis and to avoid valve migration during the implantation procedure.

### Implantation procedure

The experimental procedure consisted of induction of anaesthesia by intravenous infusion of propofol hydrochloride. Anaesthesia was maintained by inhalation of 3.0% isoflurane. Tidal volume was controlled at 8–10 mL/kg with an inspiratory rate of 13 breaths/min. Animals were placed supine on the operating table. The neck was disinfected and covered with a sterile drape. After general anaesthesia, heparin (1.5 mg/kg) was injected intravenously while the animals’ respiration, heart rate, blood pressure and blood oxygen saturation were monitored in real time.

Make a 3–4 cm incision on the right side of the neck to fully expose the right internal jugular vein and sew two 4-0 prolene (Ethicon, Inc., Raritan, NJ, USA) purse-strings to the right jugular vein. A puncture needle was inserted into the right internal jugular vein at a suitable angle. Advance the guide wire through the needle and into the vein. Typically, to ensure smooth entry into the right atrium, the guidewire should be advanced about 15–20 cm. The guidewire is then advanced across the TV into the right ventricle (RV) and further into the pulmonary artery under digital subtraction angiography and TTE guidance.

One end of the 6F pigtail catheter was inserted over the previously inserted guidewire and the catheter was slided down along the guidewire until the tip of the catheter reaches the desired depth. Preoperative RV angiography was done with a 6F pigtail catheter showing right atrium (RA) and TA. This allowed an optimal C-arm angle to be set for the operation. After gaining access into the RA through the antegrade transjugular approach under the lead of super hard guide wire, the distal end of the delivery system was bent up to make sure delivery sheath reach RV and position coaxially with the center of the pig TV under the guidance of TTE and fluoroscopy. And the tricuspid valved stent aligned with the plane of the TA.

A simple rotary system on the delivery unit was then retracted the outer casing in stages. The device was released and the stent valve unfolded. The valve was released by first releasing the hooks through the chords, adjusting the overall position of the delivery device, and then slowly releasing the outer stent, which was anchored to the leaflet by the outer stent anchor pins in conjunction with the hooks. The inner stent was released by withdrawal of the sheath, the biological leaflet begins to open and close, and the interventional valve was fully released by unlocking the inner stent delivery system. The delivery system was used to fine-tune the device position, minimizing PVL under TTE and fluoroscopic guidance. After confirmation of this, the operator continued the release of the device. Finally, the delivery system was fully retracted and the purse strings tied. RV angiography and TTE were again performed for confirmation of bioprosthesis position and whether there was paravalvular leak and/or TR. After heparin neutralisation, the neck incision was closed in layers as usual.

### Postoperative management

All animals were closely monitored postoperatively. To prevent thrombosis of the bioprosthesis, each porcine was routinely administered 4.5 mg of warfarin anticoagulant per day postoperatively and coagulation was monitored regularly with INR values maintained between 1.8 and 2.5. Antiplatelet therapy was not required as the tricuspid valve device was placed on the right cardiac venous system. Post-mortem examination of the device and cardiac tissues was performed on all animals. All animals underwent TTE before euthanasia and live animals 3 months.

### Data analysis

Hemodynamic measurements after interventional valve implantation were reported using standard descriptive statistics, and were given as mean ± standard deviation (SD).

## Results

### Intraprocedural and 3-month follow up outcomes

Transcatheter right jugular access was performed in a porcine model (n = 2), 2 animals were successfully implanted without technical difficulties. Animals were closely monitored until the end of the study. Two pigs survived for 3 months and were sacrificed per protocol. No cardiac or noncardiac complications occurred in any of the surviving animals.

### Echocardiography and fluoroscopy results

**Fig. 2:**
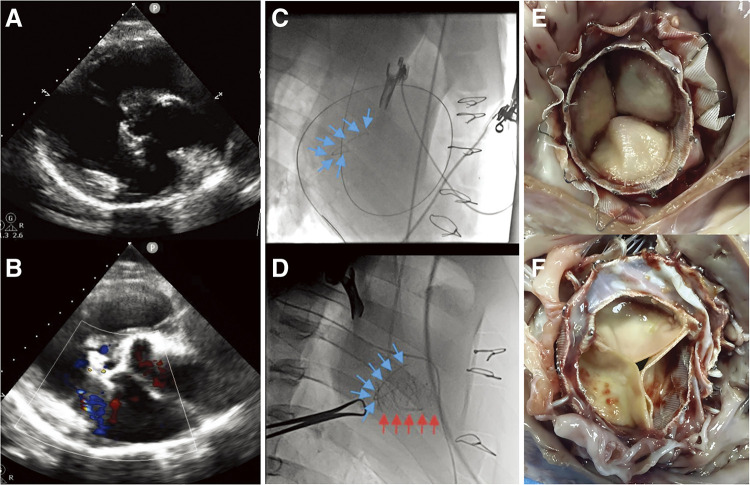
Postoperative Management. (**A** and **B**) Echocardiography: Appropriate position of the bioprosthesis, with mild central tricuspid regurgitation but no perivalvular leakage. (**C** and **D**) Fluoroscopy: transjugular tricuspid valved stent implantation. Tricuspid annulus prosthesis (blue arrows), valved stent (red arrows). (**E** and **F**) Pathology: The valved stent were well positioned on gross examination. Part of the annulus was covered by endothelial tissue. Pathology did not show any significant thrombosis, incrustation or calcified nodules in the tissue between the stent and the original TA.

Fluoroscopy and TTE results showed appropriate bioprosthesis position in 2 surviving pigs, with excellent function and mobility of the new valve (**Fig. 2**). Perioperative examination revealed no PVL and central TR in 2 pigs. No significant haemodynamic changes were observed. The mean transvalvular gradient was 1.69 ± 0.7 mmHg at 3 months. There was no PVL in either of the pigs, but one of them had a mild central TR. No right ventricular outflow tract (RVOT) obstruction, pericardial effusion, coronary artery compression, arrhythmias or atrial-ventricular conduction disturbances were observed during 3 months follow-up. Two implanted bioprostheses showed stability and normal function with no evidence of fracture or migration.

### Gross necropsy and pathology

Two animals were sacrificed for necropsy and pathology, focusing on structural integrity and device biocompatibility. The bioprostheses were well positioned on gross examination. No apparent atrial or ventricular congestion or necrosis, and no apparent atrial or ventricular damage was observed. Part of the annulus was covered by neoplastic endothelial tissue at 3 months, indicating good device biocompatibility (**Figs. 2E–2F**). Pathology did not show any thrombus, incrustation or calcified nodules in the tissue between the stent and the original TA (**Figs. 2E–2F**). In addition, the endothelium of the internal jugular vein was clean, smooth, free of thrombus, and intact without perforation.

## Discussion

TR remains a major problem for cardiologists, particularly in those who develop TR associated with RV dysfunction and right heart failure.^6)^ As a result, many of these patients are denied the option of undergoing isolated tricuspid surgery. In the last few years, with the increased understanding of TV disease and the maturation of valve technology, transcatheter interventional treatment of the tricuspid valve has emerged as an effective treatment for patients at high risk of surgical intervention. 2021 European Society of Cardiology valve management guidelines suggest that transcatheter tricuspid valve interventions (Class IIb) may be performed in nonsurgical patients in centres with expertise in tricuspid valve management.^7)^

Several transcatheter techniques have been developed experimentally and tested in animal models and clinical trial application. However, few have been used to treat TR after tricuspid valvuloplasty with ring. Interventional tricuspid valves currently under development or in clinical trials, such as the Boudjemline^8)^ (Medtronic Inc., Minneapolis, MN, USA), and Lux^9)^ (Ningbo Jenscare Biotechnology Co., Ningbo, China) valve systems, use a single standard circular design, with an inherent risk of PVL, particularly at the anterior septum.

In a study of tricuspid valve-in-ring implantation (n = 20), Aboulhosn et al.^10)^ similarly found that 75% of patients developed postoperative PVL, which occurred predominantly at sites where annular continuity was disrupted. Therefore, there was an urgent need to address the postoperative PVL problems. Our study demonstrates that tricuspid valve-in-ring implantation is achievable.

The outer valve stent is designed to match the position and shape of the dilated annulus in TR. Soft stenting is adjustable to lesion conformation for better fit to diseased annulus. The stent consists of an outer layer with two flared spurs and an inner layer with multiple inverted hooks for anchorage of the valve leaflets. To ensure that deformation of the outer stent does not affect the inner stent, the two layers are joined by a soft and airtight connection. To improve sealing and reduce PVL, the flange structure of the outer stent conforms to the top of the valve ring.

In this study, the tricuspid valve device was used in an animal model of tricuspid regurgitation that underwent tricuspid annuloplasty. The incidence of rheumatic heart disease remains high in developing countries such as China. Tricuspid annuloplasty is performed at the same time as the vast majority of operations for rheumatic left heart valve disease. As a result, there is currently a certain volume of post-tricuspid annuloplasty patients in China, and these patients undergo redo tricuspid valve surgery after recurrence of tricuspid regurgitation, which is associated with high operative mortality and poor prognosis. Therefore, this group of patients is in urgent need of less invasive tricuspid valve surgery in the future and is the main target population for our tricuspid valve device. The tricuspid device can be applied to tricuspid valves without tricuspid annulus and to tricuspid valves with bioprosthetic valves (valve-in-valve), and we have also experimented with both sets of valves and made some adjustments to the tricuspid device, as described in other papers currently being submitted.

This study has some limitations. Firstly, the number of experimental animals is very low and observational period is very short. Longer observation times are needed to check TTVDs function. However, we plan to continue this study and further optimize the tricuspid valve device, as well as conduct ongoing follow-up experiments to further validate the reliability of the tricuspid valve device. Secondly, despite the success of the animal model of TR using tricuspid annuloplasty rings, the lack of surgical expertise is still a challenge. The TTVD technique needs more practice. Thirdly, the trans-jugular approach appears to be somewhat invasive and may be difficult to perform under local anaesthesia. We are also working on the design of the transfemoral venous access and developing a transfemoral catheter.

## Conclusions

The present animal study demonstrates that implantation of this novel interventional valve device via the jugular venous route is safe and feasible. It effectively improves TR, has good manoeuvrability and stable haemodynamics after implantation. This is the first attempt at valve implantation after tricuspid valve annuloplasty with ring, which is an innovative approach. In the meantime, the transjugular route is less invasive and widely available and may become an effective alternative treatment for patients with high surgical risk of TR. Our experimental results also provide safe and effective data for the next clinical trial of this valve-in-ring device.

## Declarations

### Ethics approval and consent to participate

This study was approved by the Ethics Review Committee of Guangdong Provincial People’s Hospital (approval number: 2019-720A-1). Participant’s informed consent form is not applicable.

### Consent for publication

Not applicable.

### Funding

Huanlei Huang reports that this study was supported by the National Natural Science Foundation of China (No. 82270373), Department of Science and Technology of Guangdong Province (No. 2020B1111170011), Guangdong Basic and Applied Basic Research Foundation (No. 2019B1515120071), and High-level Hospital Construction Project of Guangdong Provincial People’s Hospital (No. 2023P-GX08).

### Author contributions

(I) Conception and design: All authors; (II) Administrative support: Huanlei Huang; (III) Provision of study materials or patients: Huanlei Huang, Lishan Zhong; (IV) Collection and assembly of data: All authors; (V) Manuscript writing: All authors; (VI) Final approval of manuscript: All authors.

### Data availability

The data that support the findings of this study are available on request from the corresponding author, upon reasonable request.

### Disclosure statement

The authors report no conflicts of interest.

## References

[1] Hahn RT Badano LP Bartko PE Tricuspid regurgitation: recent advances in understanding pathophysiology, severity grading and outcome. Eur Heart J Cardiovasc Imaging 2022; 23: 913–29. 35157070 10.1093/ehjci/jeac009

[2] Alqahtani F Berzingi CO Aljohani S Contemporary trends in the use and outcomes of surgical treatment of tricuspid regurgitation. J Am Heart Assoc 2017; 6: e007597. 29273638 10.1161/JAHA.117.007597 PMC5779056

[3] Otto CM Nishimura RA Bonow RO 2020 ACC/AHA guideline for the management of patients with valvular heart disease: a report of the American College of Cardiology/American Heart Association Joint Committee on Clinical Practice Guidelines. Circulation 2021; 143: e72–e227. 33332150 10.1161/CIR.0000000000000923

[4] Czapla J Claus I Martens T Midterm comparison between different annuloplasty techniques for functional tricuspid regurgitation. Ann Thorac Surg 2022; 114: 134–41. 34453924 10.1016/j.athoracsur.2021.07.073

[5] Shiran A Sagie A. Tricuspid regurgitation in mitral valve disease: incidence, prognostic implications, mechanism, and management. J Am Coll Cardiol 2009; 53: 401–8. 19179197 10.1016/j.jacc.2008.09.048

[6] Desai RR Vargas Abello LM Klein AL Tricuspid regurgitation and right ventricular function after mitral valve surgery with or without concomitant tricuspid valve procedure. J Thorac Cardiovasc Surg 2013; 146: 1126–1132.e10. 23010580 10.1016/j.jtcvs.2012.08.061 PMC4215162

[7] Vahanian A Beyersdorf F Praz F 2021 ESC/EACTS Guidelines for the management of valvular heart disease. Eur Heart J 2022; 43: 561–632. 34453165 10.1093/eurheartj/ehab395

[8] Boudjemline Y Agnoletti G Bonnet D Steps toward the percutaneous replacement of atrioventricular valves: an experimental study. J Am Coll Cardiol 2005; 46: 360–5. 16022968 10.1016/j.jacc.2005.01.063

[9] Lu FL Ma Y An Z First-in-man experience of transcatheter tricuspid valve replacement with lux-valve in high-risk tricuspid regurgitation patients. JACC Cardiovasc Interv 2020; 13: 1614–6. 32646711 10.1016/j.jcin.2020.03.026

[10] Aboulhosn J Cabalka AK Levi DS Transcatheter valve-in-ring implantation for the treatment of residual or recurrent tricuspid valve dysfunction after prior surgical repair. JACC Cardiovasc Interv 2017; 10: 53–63. 28057286 10.1016/j.jcin.2016.10.036

